# Pseudo-Weak-*R*_0_ Algebras

**DOI:** 10.1155/2014/352381

**Published:** 2014-05-04

**Authors:** Yong Lin Liu, Xiaobo Cai

**Affiliations:** ^1^Department of Mathematics and Computer, Wuyi University, Wuyishan, Fujian 354300, China; ^2^Department of Computer Science and Technology, Chuxiong Normal University, Chuxiong, Yunnan 675000, China

## Abstract

A positive answer to the open problem of Iorgulescu on extending weak-*R*_0_ algebras and *R*_0_-algebras to the noncommutative forms is given. We show that pseudo-weak-*R*_0_ algebras are categorically isomorphic to pseudo-IMTL algebras and that pseudo-*R*_0_ algebras are categorically isomorphic to pseudo-NM algebras. Some properties, the noncommutative forms of the properties in weak-*R*_0_ algebras and *R*_0_-algebras, are investigated. The simplified axiom systems of pseudo-weak-*R*_0_ algebras and pseudo-*R*_0_ algebras are obtained.

## 1. Introduction

It is well known that certain information processing, especially inferences based on certain information, is based on classical two-valued logic. Due to strict and complete logical foundation (classical logic), making inferences about certain information can be done with high confidence levels. Thus, it is natural and necessary to attempt to establish some rational logic system as the logical foundation for uncertain information processing. It is evident that this kind of logic cannot be two-valued logic itself but might form a certain extension of two-valued logic. Various kinds of nonclassical logic systems have therefore been extensively researched in order to construct natural and efficient inference systems to deal with uncertainty.

In recent years, motivated by both theory and application, the study of t-norm-based logic systems and the corresponding pseudo-logic systems has become of greater focus in the field of logic. Here, t-norm-based logical investigations preceded the corresponding algebraic investigations, and in the case of pseudo-logic systems, algebraic development preceded the corresponding logical development.

A noncommutative generalization of reasoning can be found, for example, in psychological processes. In clinical medicine on behalf of transplantation of human organs, an experiment was performed in which the same two questions have been posed to two groups of interviewed people as follows. (1) Do you agree to donate your organs for medical transplantation after your death? (2) Do you agree to accept organs of a donor if you need them? When the order of questions was changed in the second group, the number of positive answers here was much higher than in the first group.

The following reviews some situation concerning some important logic algebras and the corresponding pseudo-logic algebras. BCK- and BCI-algebras were introduced by Imai and Iseki [1] and have been extensively investigated by many researchers. Georgescu and Iorgulescu [2] introduced the notion of a pseudo-BCK-algebra as a noncommutative generalization of a BCK-algebra. Liu et al. [3] investigated the theory of pseudo-BCK-algebras. MV-algebras were introduced by Chang in [4] as an algebraic tool to study the infinitely valued logic of Lukasiewicz. Georgescu and Iorgulescu [5] introduced pseudo-MV-algebras which are a noncommutative generalization of MV-algebras. The notion of BL-algebras was introduced by Hajek [6] as the algebraic structures for his basic logic. Georgescu and Iorgulescu [7] introduced the notion of pseudo-BL-algebras by dropping commutative axioms in BL-algebras. Di Nola et al. [8, 9], Zhang and Fan [10], and Zhan et al. [11] investigated in detail the theory of pseudo-BL-algebras. MTL-algebras [12] are the algebraic structures for Esteva-Godo monoidal t-norm-based logic, a many-valued propositional calculus that formalizes the structure of the real unit interval [0,1], induced by a left-continuous t-norm. Flondor et al. [13] presented pseudo-MTL-algebras as a noncommutative generalization of MTL-algebras.

IMTL-algebras [12] are the algebraic counterpart for involutive monoidal t-norm logic, an extension of MTL-algebras. NM-algebras [12] are the algebraic counterpart for nilpotent minimum logic, an extension of IMTL-algebras. *R*_0_-algebras were introduced by Wang [14] as the algebraic structure for his formal deductive system L* of fuzzy propositional calculus. Weak-*R*_0_-algebras [14] are the generalization of *R*_0_-algebras. In the recent years, the research on *R*_0_-algebras has attracted more and more attention [15]. In [16], Iorgulescu proposed one open problem (open problem 2.14 of [16]).

*Problem*. Recall that the IMTL-algebras, introduced in 2001 by Esteva and Godo, are categorically isomorphic to weak-*R*_0_ algebras, introduced in 1997 by Wang, and that NM-algebras are categorically isomorphic to *R*_0_-algebras, introduced also in 1997 by Wang. Extend weak-*R*_0_ algebras and *R*_0_-algebras to the noncommutative case.

In this paper, we extend weak-*R*_0_ algebras and *R*_0_-algebras to the noncommutative forms, called pseudo-weak-*R*_0_ algebras and pseudo-*R*_0_ algebras. We show that pseudo-weak-*R*_0_ algebras are categorically isomorphic to pseudo-IMTL-algebras and that pseudo-*R*_0_ algebras are categorically isomorphic to pseudo-NM algebras. Some properties, the noncommutative forms of the properties in weak-*R*_0_ algebras and *R*_0_-algebras, are investigated. Furthermore, we discuss the simplified axiom systems of pseudo-weak-*R*_0_ algebras and pseudo-*R*_0_ algebras.

## 2. Preliminaries

We recall some definitions and results which will be used in the sequel.

**Definition 1 (see [12]).**

An MTL-algebra (or a weak-BL-algebra) is a structure (*A*, ∨, ∧, ⊙, →, 0,1) of type (2,2, 2,2, 0,0) such that for all *x*, *y*, *z* ∈ *A*

(B1) (*A*, ∨, ∧, 0,1) is a bounded lattice,
(B2) (*A*, ⊙, 1) is a monoid,
(B3) *x*⊙*y* ≤ *z* if and only if *x* ≤ *y* → *z*,
(B4) (*x* → *y*)∨(*y* → *x*) = 1.

**Definition 2 (see [12]).**

An IMTL- (involutive MTL-) algebra is an MTL-algebra satisfying the following additional condition:

(B5) *x* ^−−^ = *x*,

where *x* ^−^ = *x* → 0.

A WNM- (weak nilpotent minimum-) algebra is an MTL-algebra satisfying the following additional condition:

(B6) (*x*⊙*y*)^−^∨((*x*∧*y*)→(*x*⊙*y*)) = 1.

An NM- (nilpotent minimum-) algebra is an IMTL-algebra satisfying condition (B6).

**Definition 3 (see [13]).**

A pseudo-MTL algebra (or a weak-pseudo-BL algebra) is a structure (*A*, ∨, ∧, ⊙, →, ⇝, 0,1) of type (2,2, 2,0, 0) such that for all *x*, *y*, *z* ∈ *A*

(pB1) (*A*, ∨, ∧, 0,1) is a bounded lattice,
(pB2) (*A*, ⊙, 1) is a monoid,
(pB3) *x*⊙*y* ≤ *z* if and only if *x* ≤ *y* → *z* if and only if *y* ≤ *x*⇝*z*,
(pB4) (*x* → *y*)∨(*y* → *x*) = (*x*⇝*y*)∨(*y*⇝*x*) = 1.

**Definition 4 (see [16, 17]).**

A pseudo-IMTL (pseudo-involutive MTL) algebra is a pseudo-MTL algebra satisfying the following additional condition:

(pB5) *x* ^~−^ = *x* ^−~^ = *x*,

where *x* ^−^ = *x* → 0 and *x* ^~^ = *x*⇝0,

A pseudo-WNM (pseudo-weak nilpotent minimum) algebra is a pseudo-MTL algebra satisfying the following additional condition:

(pB6) (*x*⊙*y*)^−^∨((*x*∧*y*)→(*x*⊙*y*)) = (*x*⊙*y*)^~^∨((*x*∧*y*)⇝(*x*⊙*y*)) = 1,

where *x* ^−^ = *x* → 0 and *x* ^~^ = *x*⇝0.

A pseudo-NM (pseudo-nilpotent minimum) algebra is a pseudo-IMTL algebra satisfying condition (pB6).

**Definition 5 (see [14, 18]).**

Let *M* be a (¬, ∧, ∨, →)-type algebra, where ¬ is a unary operation and ∧, ∨, and → are binary operations. If there is a partial ordering ≤ on *M*, such that (*M*, ≤) is a bounded distributive lattice, ∧, ∨ are infimum and supremum operations with respect to ≤, ¬ is an order-reversing involution with respect to ≤, and the following conditions hold for any *a*, *b*, *c* ∈ *M*:

(R1) ¬*a* → ¬*b* = *b* → *a*,
(R2) 1 → *a* = *a*, *a* → *a* = 1,
(R3) *b* → *c* ≤ (*a* → *b*)→(*a* → *c*),
(R4) *a* → (*b* → *c*) = *b* → (*a* → *c*),
(R5) *a* → (*b*∨*c*) = (*a* → *b*)∨(*a* → *c*), *a* → (*b*∧*c*) = (*a* → *b*)∧(*a* → *c*),

where 1 is the largest element of *M*, then one calls *M* a weak-*R*_0_ algebra.

An *R*_0_ algebra *M* is a weak-*R*_0_ algebra satisfying the following additional condition:

(R6) (*a* → *b*)∨((*a* → *b*)→(¬*a*∨*b*)) = 1.

## 3. Pseudo-Weak-*R*_0_ Algebras and Pseudo-*R*_0_ Algebras

We introduce the notions of pseudo-weak-*R*_0_ algebras and pseudo-*R*_0_ algebras. They are noncommutative forms of weak-*R*_0_ algebras and *R*_0_-algebras. Some of their properties are investigated.

**Definition 6 .**

A pseudo-weak-*R*_0_ algebra is a structure (*A*, ∧, ∨, →, ⇝, ^−^, ^~^, 0,1) such that (*A*, ∧, ∨, 0,1) is a bounded distributive lattice, ^−^ and ^~^ are order-reversing pseudo-involution (i.e., if *x* ≤ *y*, then *y* ^−^ ≤ *x* ^−^ and *y* ^~^ ≤ *x* ^~^; *x* ^~−^ = *x* ^−~^ = *x*), and the following axioms hold for any *x*, *y*, *z* ∈ *A*:

 (pR1) *x* → *y* = *y* ^−^⇝*x* ^−^,  *x*⇝*y* = *y* ^~^ → *x* ^~^,
 (pR2*) 1 → *x* = 1⇝*x* = *x*; *x* → *x* = *x*⇝*x* = 1,
 (pR3) *x* → *y* ≤ (*z* → *x*)→(*z* → *y*), *x*⇝*y* ≤ (*z*⇝*x*)⇝(*z*⇝*y*),
 (pR4) *x* → (*y*⇝*z*) = *y*⇝(*x* → *z*),
 (pR5*) *x* → (*y*∨*z*) = (*x* → *y*)∨(*x* → *z*), *x*⇝(*y*∨*z*) = (*x*⇝*y*)∨(*x*⇝*z*);  *x* → (*y*∧*z*) = (*x* → *y*)∧(*x* → *z*), *x*⇝(*y*∧*z*) = (*x*⇝*y*)∧(*x*⇝*z*).

A pseudo-*R*_0_ algebra *A* is a pseudo-weak-*R*_0_ algebra satisfying the following additional axiom:

 (pR6) (*x* → *y*)∨((*x* → *y*)⇝(*x* ^−^∨*y*)) = (*x*⇝*y*)∨((*x*⇝*y*)→(*x* ^~^∨*y*)) = 1.

**Remark 7 .**

(i) Comparing Definition 6 with Definition 5, the notions of pseudo-weak-*R*_0_ algebras and pseudo-*R*_0_ algebras are the noncommutative forms of weak-*R*_0_ algebras and *R*_0_-algebras, respectively.

(ii) The operations ^−^ and ^~^ in Definition 6 are not primary (please see Proposition 10 (2)).

(iii) As we will see in Proposition 10 (3), (10), and (14), the following axioms in Definition 6 follow from the other axioms:

- the distributivity of bounded lattice (*A*, ∧, ∨, 0,1),
- *x* → *x* = *x*⇝*x* = 1,
- *x* → (*y*∧*z*) = (*x* → *y*)∧(*x* → *z*), *x*⇝(*y*∧*z*) = (*x*⇝*y*)∧(*x*⇝*z*).

Hence, we have the following simplified definition. In what follows, we will use the following definition.

**Definition 8 .**

A pseudo-weak-*R*_0_ algebra is a structure (*A*, ∧, ∨, →, ⇝, ^−^, ^~^, 0,1) satisfying the following:

(pL1) (*A*, ∧, ∨, 0,1) is a bounded lattice,
(pL2) if *x* ≤ *y*, then *y* ^−^ ≤ *x* ^−^ and *y* ^~^ ≤ *x* ^~^,
(pL3) *x* ^~−^ = *x* ^−~^ = *x*,
(pR1) *x* → *y* = *y* ^−^⇝*x* ^−^, *x*⇝*y* = *y* ^~^ → *x* ^~^,
(pR2) 1 → *x* = 1⇝*x* = *x*,
(pR3) *x* → *y* ≤ (*z* → *x*)→(*z* → *y*), *x*⇝*y* ≤ (*z*⇝*x*)⇝(*z*⇝*y*),
(pR4) *x* → (*y*⇝*z*) = *y*⇝(*x* → *z*),
(pR5) *x* → (*y*∨*z*) = (*x* → *y*)∨(*x* → *z*), *x*⇝(*y*∨*z*) = (*x*⇝*y*)∨(*x*⇝*z*).

A pseudo-*R*_0_ algebra *A* is a pseudo-weak-*R*_0_ algebra satisfying the following additional axiom:

(pR6) (*x* → *y*)∨((*x* → *y*)⇝(*x* ^−^∨*y*)) = (*x*⇝*y*)∨((*x*⇝*y*)→(*x* ^~^∨*y*)) = 1.

**Example 9 .**

Let *A* = [0,1], and define operations ∨, ∧, ^−^, ^~^ on *A* as follows:

(1) $$\begin{array}{c} x\vee y=\max\left\{x,y\right\},\;\;x\wedge y=\min\left\{x,y\right\},\\ x^{-}=\sqrt{1-x,}\;\;x^{\sim }=1-x^{2}. \end{array}$$

Then, (*A*, ∧, ∨,^−^,^~^, 0,1) is a bounded lattice satisfying *x* ^−~^ = *x* ^~−^ = *x* for any *x* ∈ *A*. Define operations → and ⇝ as pseudo-Lukasiewicz implication on *A*:

(2) $$\begin{array}{c} x⟶y=1\wedge \left(x^{-}+y\right),\;\;x⇝y=1\wedge \left(x^{\sim }+y\right). \end{array}$$

By routine calculations, (*A*, ∧, ∨, →, ⇝, ^−^, ^~^, 0,1) is a pseudo-weak-*R*_0_ algebra.

**Proposition 10 .**

In a pseudo-weak-*R*_0_ algebra, the following properties hold:

1. 0^~^ = 0^−^ = 1, 1^~^ = 1^−^ = 0,
2. *x* ^−^ = *x* → 0, *x* ^~^ = *x*⇝0,
3. *x*⇝*x* = *x* → *x* = 1,
4. *x* ≤ *y if and only if x*⇝*y* = 1* if and only if x* → *y* = 1,
5. (⋀_*i*∈*I*_ *x*_*i*_)^~^ = ⋁_*i*∈*I*_ *x*_*i*_^~^, (⋀_*i*∈*I*_ *x*_*i*_)^−^ = ⋁_*i*∈*I*_ *x*_*i*_^−^,* whenever the arbitrary meets and unions exist,*
6. (⋁_*i*∈*I*_ *x*_*i*_)^~^ = ⋀_*i*∈*I*_ *x*_*i*_^~^, (⋁_*i*∈*I*_ *x*_*i*_)^−^ = ⋀_*i*∈*I*_ *x*_*i*_^−^,* whenever the arbitrary meets and unions exist,*
7. *if x* ≤ *y*,* then z*⇝*x* ≤ *z*⇝*y and z* → *x* ≤ *z* → *y*,
8. *if x* ≤ *y*,* then y*⇝*z* ≤ *x*⇝*z and y* → *z* ≤ *x* → *z*,
9. (*x*∧*y*)⇝*z* = (*x*⇝*z*)∨(*y*⇝*z*), (*x*∧*y*) → *z* = (*x* → *z*)∨(*y* → *z*),
10. *x*⇝(*y*∧*z*) = (*x*⇝*y*)∧(*x*⇝*z*), *x* → (*y*∧*z*) = (*x* → *y*)∧(*x* → *z*),
11. (*x*∨*y*)⇝*z* = (*x*⇝*z*)∧(*y*⇝*z*), (*x*∨*y*) → *z* = (*x* → *z*)∧(*y* → *z*),
12. *x* ^~^∨*y* ≤ *x*⇝*y*, *x* ^−^∨*y* ≤ *x* → *y*,
13. *x*⇝*y* ≤ (*y*⇝*z*)→(*x*⇝*z*), *x* → *y* ≤ (*y* → *z*)⇝(*x* → *z*),
14. (*A*, ∧, ∨, 0,1)* is a bounded distributive lattice*.

**Proof**

(1) Since 0 ≤ 1^~^ and 0 ≤ 1^−^, by (L3) and (L2), 1 = 1^~−^ ≤ 0^−^ and 1 = 1^−~^ ≤ 0^~^, and hence 0^~^ = 0^−^ = 1. 1^~^ = 1^−^ = 0 follows similarly.

(2) By (1), (pR1), and (pR2), *x* ^−^ = 1⇝*x* ^−^ = *x* ^−~^ → 1^~^ = *x* → 0, *x* ^~^ = 1 → *x* ^~^ = *x* ^~−^⇝1^−^ = *x*⇝0.

(3) By (pR1) and (pR2), 0⇝0 = 0^~^ → 0^~^ = 1 → 1 = 1. By (pR3) and (L3), 0⇝0 ≤ (*x* ^−^⇝0)⇝(*x* ^−^⇝0); that is, 1 ≤ *x*⇝*x*, and so *x*⇝*x* = 1. The second equality follows similarly.

(4) If *x* → *y* = 1, by (pR1), (pR3), and (pR2), *x* = 1 → *x* = *x* ^−^⇝0≤(*y* ^−^⇝*x* ^−^)⇝(*y* ^−^⇝0) = (*x* → *y*)⇝(1 → *y*) = 1⇝*y* = *y*. If *x* ≤ *y*, by (pR5) and (3), *x* → *y* = *x* → (*x*∨*y*) = (*x* → *x*)∨(*x* → *y*) = 1∨(*x* → *y*) = 1. Similarly, we have *x* ≤ *y* if and only if *x*⇝*y* = 1.

(5) Since ⋀_*i*∈*I*_ *x*_*i*_ ≤ *x*_*i*_ for each *i* ∈ *I*, by (L2), *x*_*i*_^~^ ≤ (⋀_*i*∈*I*_ *x*_*i*_)^~^ for each *i* ∈ *I*, and so ⋁_*i*∈*I*_ *x*_*i*_^~^ ≤ (⋀_*i*∈*I*_ *x*_*i*_)^~^. Since *x*_*i*_^~^ ≤ ⋁_*i*∈*I*_ *x*_*i*_^~^ for each *i* ∈ *I*, by (L2) and (L3), (⋁_*i*∈*I*_ *x*_*i*_^~^)^−^ ≤ *x*_*i*_ for each *i* ∈ *I*, and so (⋁_*i*∈*I*_ *x*_*i*_^~^)^−^ ≤ ⋀_*i*∈*I*_ *x*_*i*_, (⋀_*i*∈*I*_ *x*_*i*_)^~^ ≤ ⋁_*i*∈*I*_ *x*_*i*_^~^. The second equality follows similarly.

(6) Similarly.

(7) If *x* ≤ *y*, by (pR5), *z*⇝*y* = *z*⇝(*x*∨*y*) = (*z*⇝*x*)∨(*z*⇝*y*), and so *z*⇝*x* ≤ *z*⇝*y*. Similarly, *z* → *x* ≤ *z* → *y*.

(8) If *x* ≤ *y*, then *y* ^−^ ≤ *x* ^−^. By (pR1), *y* → *z* = *z* ^−^⇝*y* ^−^ ≤ *z* ^−^⇝*x* ^−^ = *x* → *z*. Similarly, *y*⇝*z* ≤ *x*⇝*z*.

(9) By (pR1), (5), and (pR5), (*x*∧*y*) → *z* = *z* ^−^⇝(*x*∧*y*)^−^ = *z* ^−^⇝(*x* ^−^∨*y* ^−^) = (*z* ^−^⇝*x* ^−^)∨(*z* ^−^⇝*y* ^−^) = (*x* → *z*)∨(*y* → *z*). Similarly, (*x*∧*y*)⇝*z* = (*x*⇝*z*)∨(*y*⇝*z*).

(10) By (4), (pR1), and (pR4), for any *u*, *u* ≤ *x* → (*y*∧*z*) if and only if *u* → (*x* → (*y*∧*z*)) = 1 if and only if *u* → ((*y*∧*z*)^−^⇝*x* ^−^) = 1 if and only if (*y*∧*z*)^−^⇝(*u* → *x* ^−^) = 1 if and only if (*u* → *x* ^−^)^~^ → (*y*∧*z*) = 1 if and only if (*u* → *x* ^−^)^~^ ≤ *y*∧*z* if and only if (*u* → *x* ^−^)^~^ ≤ *y* and (*u* → *x* ^−^)^~^ ≤ *z* if and only if (*u* → *x* ^−^)^~^ → *y* = 1 and (*u* → *x* ^−^)^~^ → *z* = 1 if and only if *u* ≤ *x* → *y* and *u* ≤ *x* → *z* if and only if *u* ≤ (*x* → *y*)∧(*x* → *z*). The second equality follows similarly.

(11) By (10), (*x*∨*y*) → *z* = *z* ^−^⇝(*x*∨*y*)^−^ = *z* ^−^⇝(*x* ^−^∧*y* ^−^) = (*z* ^−^⇝*x* ^−^)∧(*z* ^−^⇝*y* ^−^) = (*x* → *z*)∧(*y* → *z*). Similarly, (*x*∨*y*)⇝*z* = (*x*⇝*z*)∧(*y*⇝*z*).

(12) Since *x* → *y* ≥ *x* → 0 = 1⇝*x* ^−^ = *x* ^−^ and *x* → *y* ≥ 1 → *y* = *y*, we have *x* ^−^∨*y* ≤ *x* → *y*. Similarly, *x* ^~^∨*y* ≤ *x*⇝*y*.

(13) By (pR4) and (pR3), (*x* → *y*)→((*y* → *z*)⇝(*x* → *z*)) = (*y* → *z*)⇝((*x* → *y*)→(*x* → *z*)) = 1. Hence, *x* → *y* ≤ (*y* → *z*)⇝(*x* → *z*). Similarly, we have the second inequality.

(14) Obviously, (*x*∧*y*)∨(*x*∧*z*) ≤ *x*∧(*y*∨*z*); by (pR5), (10), and (4),

(3) (x∧(y∨z))⇝((x∧y)∨(x∧z)) =((x∧(y∨z))⇝(x∧y))  ∨((x∧(y∨z))  ⇝(x∧z)) =[((x∧(y∨z))⇝x)∧((x∧(y∨z))⇝y)]  ∨[((x∧(y∨z))⇝x)∧((x∧(y∨z))⇝z)] =((x∧(y∨z))⇝y)∨((x∧(y∨z))⇝z) =(x∧(y∨z))⇝(y∨z) =1.

Hence, *x*∧(*y*∨*z*))≤(*x*∧*y*)∨(*x*∧*z*).

Next, we continue to investigate the properties of pseudo-weak-*R*_0_ algebras and pseudo-*R*_0_ algebras, which are needed in the sequel.

**Proposition 11 .**

In a pseudo-weak-*R*_0_ algebra, the following properties hold:

(15) *x*⇝*y* ≤ *x*∨*z*⇝*y*∨*z*, *x* → *y* ≤ *x*∨*z* → *y*∨*z*,
(16) *x*⇝*y* ≤ *x*∧*z*⇝*y*∧*z*, *x* → *y* ≤ *x*∧*z* → *y*∧*z*,
(17) (*x*⇝*y*)≤(*x*⇝*z*)∨(*z*⇝*y*), (*x* → *y*)≤(*x* → *z*)∨(z → *y*),
(18) (*x*⇝*y*)∨(*y*⇝*x*) = (*x* → *y*)∨(*y* → *x*) = 1,
(19) *x* ≤ (*x* → *y*)⇝*y*, *x* ≤ (*x*⇝*y*) → *y*,
(20) *x* → *y* = ((*x* → *y*)⇝*y*) → *y*, *x*⇝*y* = ((*x*⇝*y*) → *y*)⇝*y*,
(21) *x* → (*y* → *x*) = *x*⇝(*y*⇝*x*) = *x*⇝(*y* → *x*) = *x* → (*y*⇝*x*) = 1,
(22) *x* ^−^ → (*x* → *y*) = *x* ^~^⇝(*x*⇝*y*) = *x* ^−^⇝(*x* → *y*) = *x* ^~^ → (*x*⇝*y*) = 1,
(23) *y* ≤ (*x*⇝*y*)∧(*x* → *y*),
(24) *x*∨*y* = ((*x*⇝*y*) → *y*)∧((*y*⇝*x*) → *x*) = ((*x* → *y*)⇝*y*)∧((*y* → *x*)⇝*x*),
(25) (*x*∨*y*) → *x* = *y* → *x*, (*x*∨*y*)⇝*x* = *y*⇝*x*,
(26) *x* → (*x*∧*y*) = *x* → *y*, *x*⇝(*x*∧*y*) = *x*⇝*y*,
(27) *x* ≤ *y* ^−^ *if and only if y* ≤ *x* ^~^,
(28) *x* → *y* ^~^ = *y*⇝*x* ^−^, *x*⇝*y* ^−^ = *y* → *x* ^~^,
(29) (*x* → *y* ^−^)^~^ = (*y*⇝*x* ^~^)^−^.

**Proof**

(15) Since *z*⇝(*y*∨*z*) = 1, *x*⇝*y* ≤ *x*⇝(*y*∨*z*) = (*x*⇝(*y*∨*z*))∧(*z*⇝(*y*∨*z*)) = (*x*∨*z*)⇝(*y*∨*z*). The second inequality follows similarly.

(16) Since (*x*∧*z*)⇝*z* = 1, *x*⇝*y* ≤ (*x*∧*z*)⇝*y* = ((*x*∧*z*)⇝*y*)∧((*x*∧*z*)⇝*z*) = (*x*∧*z*)⇝(*y*∧*z*). The second inequality follows similarly.

(17) Consider *x*⇝*y* ≤ (*x*∨*z*)⇝(*y*∨*z*) = ((*x*∨*z*)⇝*y*)∨((*x*∨*z*)⇝*z*) = ((*x*⇝*y*)∧(*z*⇝*y*))∨ ((*x*⇝*z*)∧(*z*⇝*z*))≤(*x*⇝*z*)∨(*z*⇝*y*). The second inequality follows similarly.

(18) Consider 1 = (*x*∨*y*)⇝(*x*∨*y*) = ((*x*∨*y*)⇝*x*)∨((*x*∨*y*)⇝*y*) = ((*x*⇝*x*)∧(*y*⇝*x*))∨((*x*⇝*y*)∧(*y*⇝*y*)) = (*x*⇝*y*)∨(*y*⇝*x*). The second equality follows similarly.

(19) Consider *x* → ((*x* → *y*)⇝*y*) = (*x* → *y*)⇝(*x* → *y*) = 1 and *x*⇝((*x*⇝*y*) → *y*) = (*x*⇝*y*)→(*x*⇝*y*) = 1.

(20) Since (((*x* → *y*)⇝*y*) → *y*)⇝(*x* → *y*) ≥ *x* → ((*x* → *y*)⇝*y*) = (*x* → *y*)⇝(*x* → *y*) = 1 and (*x* → *y*)⇝(((*x* → *y*)⇝*y*) → *y*) = ((*x* → *y*)⇝*y*)→((*x* → *y*)⇝*y*) = 1, then ((*x* → *y*)⇝*y*) → *y* = (*x* → *y*). Similarly, we have ((*x*⇝*y*) → *y*)⇝*y* = *x*⇝*y*.

(21) *x* → (*y* → *x*) = 1 if and only if *x*⇝(*y* → *x*) = 1 if and only if *y* → (*x*⇝*x*) = 1. The rest follow similarly.

(22) Consider *x* ^−^ → (*x* → *y*) ≥ 0 → *y* = 1. The rest follow similarly.

(23) Consider *y* = 1⇝*y* ≤ *x*⇝*y* and *y* = 1 → *y* ≤ *x* → *y*.

(24) Since *x*⇝((*x*⇝*y*) → *y*) = (*x*⇝*y*)→(*x*⇝*y*) = 1 and *y*⇝((*x*⇝*y*) → *y*) = (*x*⇝*y*)→(*y*⇝*y*) = 1, then *x*, *y* ≤ (*x*⇝*y*) → *y*, and so *x*∨*y* ≤ (*x*⇝*y*) → *y*. Similarly, *x*∨*y* ≤ (*y*⇝*x*) → *x*. Hence, *x*∨*y* ≤ ((*x*⇝*y*) → *y*)∧((*y*⇝*x*) → *x*). On the other hand, by (9), (pR5), (20), and (18),

(4) [((x⇝y)⟶y)∧((y⇝x)⟶x)]⇝(x∨y) =[((x⇝y)⟶y)⇝(x∨y)]  ∨[((y⇝x)⟶x)⇝(x∨y)] =(((x⇝y)⟶y)⇝x)∨(((x⇝y)⟶y)⇝y)  ∨(((y⇝x)⟶x)⇝x)∨(((y⇝x)⟶x)⇝y) =(((x⇝y)⟶y)⇝x)∨(x⇝y)∨(y⇝x)    ∨(((y⇝x)⟶x)⇝y) =1.

The second equality has a similar proof.

(25) Consider (*x*∨*y*) → *x* = (*x* → *x*)∧(*y* → *x*) = *y* → *x*, (*x*∨*y*)⇝*x* = (*x*⇝*x*)∧(*y*⇝*x*) = *y*⇝*x*.

(26) Consider  *x* → (*x*∧*y*) = (*x* → *x*)∧(*x* → *y*) = *x* → *y*, *x*⇝(*x*∧*y*) = (*x*⇝*x*)∧(*x*⇝*y*) = *x*⇝*y*.

(27) *x* ≤ *y* ^−^ if and only if *x*⇝*y* ^−^ if and only if *y* → *x* ^~^ = 1 if and only if *y* ≤ *x* ^~^.

(28) Consider*x* → *y* ^~^ = *y* ^~−^⇝*x* ^−^ = *y*⇝*x* ^−^, *x*⇝*y* ^−^ = *y* ^−~^ → *x* ^~^ = *y* → *x* ^~^.

(29) For each *u*, (*x* → *y* ^−^)^~^ ≤ *u* if and only if *u* ^−^ ≤ *x* → *y* ^−^ if and only if *u* ^−^⇝(*x* → *y* ^−^) = 1 if and only if *x* → (*u* ^−^⇝*y* ^−^) = 1 if and only if *x* → (*y* → *u*) = 1 if and only if *x*⇝(*y* → *u*) = 1 if and only if *y* → (*x*⇝*u*) = 1 if and only if *y*⇝(*x*⇝*u*) = 1 if and only if *y*⇝(*u* ^~^ → *x* ^~^) = 1 if and only if *u* ^~^ → (*y*⇝*x* ^~^) = 1 if and only if *u* ^~^ ≤ *y*⇝*x* ^~^ if and only if (*y*⇝*x* ^~^)^−^ ≤ *u*.

In a pseudo-weak-*R*_0_ algebra (pseudo-*R*_0_ algebra) *A*, we define a binary operation ⊙ as follows, for any *x*, *y* ∈ *A*:

(30) *x*⊙*y* = (*x*→*y* ^−^)^~^ = (*y*⇝*x* ^~^)^−^.

**Proposition 12 .**

In a pseudo-weak-*R*_0_ algebra, the following properties hold:

(31) *x* → *y* = (*x*⊙*y* ^~^)^−^, *x*⇝*y* = (*y* ^−^⊙*x*)^~^,
(32) (*x*⊙*y*)⊙*z* = *x*⊙(*y*⊙*z*),
(33) 1⊙*x* = *x*⊙1 = *x*,
(34) *x*⊙*y* ≤ *z* *if and only if x* ≤ *y* → *z* *if and only if y* ≤ *x*⇝*z*,
(35) *x*⊙(*x*⇝*y*) ≤ *y* ≤ *x*⇝(*x*⊙*y*), (*x* → *y*)⊙*x* ≤ *y* ≤ *x* → (*y*⊙*x*),
(36) *x*⊙(*x*⇝*y*) ≤ *x* ≤ *y*⇝(*y*⊙*x*), (*x* → *y*)⊙*x* ≤ *x* ≤ *y* → (*x*⊙*y*),
(37) * if x* ≤ *y*,* then x*⊙*z* ≤ *y*⊙*z and z*⊙*x* ≤ *z*⊙*y*,
(38) *x*⊙(*x*⇝*y*) ≤ *x*∧*y*, (*x* → *y*)⊙*x* ≤ *x*∧*y*,
(39) *x*⊙0 = 0⊙*x* = 0,
(40) *x*⊙(⋁_*i*∈*I*_ *x*_*i*_) = ⋁_*i*∈*I*_(*x*⊙*x*_*i*_), (⋁_*i*∈*I*_ *x*_*i*_)⊙*x* = ⋁_*i*∈*I*_(*x*_*i*_⊙*x*),* whenever the arbitrary unions exist*,
(41) (*x*⊙*y*) → *z* = *x* → (*y* → *z*), (*y*⊙*x*)⇝*z* = *x*⇝(*y*⇝*z*),
(42) *y*⇝(⋀_*i*∈*I*_ *x*_*i*_) = ⋀_*i*∈*I*_(*y*⇝*x*_*i*_), *y* → (⋀_*i*∈*I*_ *x*_*i*_) = ⋀_*i*∈*I*_(*y* → *x*_*i*_),* whenever the arbitrary meets exist*,
(43) (⋁_*i*∈*I*_ *x*_*i*_)⇝*y* = ⋀_*i*∈*I*_(*x*_*i*_⇝*y*), (⋁_*i*∈*I*_ *x*_*i*_) → *y* = ⋀_*i*∈*I*_(*x*_*i*_ → *y*),* whenever the arbitrary unions and meets exist*,
(44) *x*⊙*x* ^~^ = *x* ^−^⊙*x* = 0,
(45) *x*⊙*y* ≤ *x*∧*y* ≤ *x*, *y*,
(46) *x*∨(*y*⊙*z*)≥(*x*∨*y*)⊙(*x*∨*z*),
(47) *x* → *y* ≤ (*x*⊙*z*)→(*y*⊙*z*), *x*⇝*y* ≤ (*z*⊙*x*)⇝(*z*⊙*y*),
(48) *x*⊙(*y* → *z*) ≤ *y* → (*x*⊙*z*), (*y*⇝*z*)⊙*x* ≤ *y*⇝(*z*⊙*x*).

**Proof**

(31) Straightforward.

(32) By (pR1) and (pR4), (*x*⊙*y*)⊙*z* = (*x* → *y* ^−^)^~^⊙*z* = ((*x* → *y* ^−^)^~^ → *z* ^−^)^~^ = (*z* ^−−^⇝(*x* → *y* ^−^))^~^ = (*x* → (*z* ^−−^⇝*y* ^−^))^~^ = (*x* → (*y* → *z* ^−^))^~^ and *x*⊙(*y*⊙*z*) = *x*⊙(*y* → *z* ^−^)^~^ = (*x* → (*y* → *z* ^−^))^~^.

(33) Consider1⊙*x* = (1 → *x* ^−^)^~^ = *x* ^−~^ = *x*, *x*⊙1 = (*x* → 1^−^)^~^ = (*x* → 0)^~^ = *x* ^−~^ = *x*.

(34) Consider*x*⊙*y* ≤ *z*⇔(*x* → *y* ^−^)^~^ ≤ *z*⇔*z* ^−^ ≤ *x* → *y* ^−^⇔*z* ^−^⇝(*x* → *y* ^−^) = 1⇔*x* → (*z* ^−^⇝*y* ^−^) = 1⇔*x* → (*y* → *z*) = 1⇔*x* ≤ *y* → *z*. And

Consider *x*⊙*y* ≤ *z*⇔(*y*⇝*x* ^~^)^−^ ≤ *z*⇔*z* ^~^ ≤ *y*⇝*x* ^~^⇔*z* ^~^ → (*y*⇝*x* ^~^) = 1⇔*y*⇝(*z* ^~^ → *x* ^~^) = 1⇔*y*⇝(*x*⇝*z*) = 1⇔*y* ≤ *x*⇝*z*.

(35) Since *x*⇝*y* ≤ *x*⇝*y*, by (34), *x*⊙(*x*⇝*y*) ≤ *y*. Since *x*⊙*y* ≤ *x*⊙*y*, by (34), *y* ≤ *x*⇝(*x*⊙*y*). The second inequality follows similarly.

(36) Since *x* → *y* ≤ 1 = *x* → *x*, we have (*x* → *y*)⊙*x* ≤ *x*. Since *x*⊙*y* ≤ *x*⊙*y*, we have *x* ≤ *y* → (*x*⊙*y*). The second inequality follows similarly.

(37) *x* ≤ *y*⇒*y* → *z* ^−^ ≤ *x* → *z* ^−^⇒(*x* → *z* ^−^)^~^ ≤ (*y* → *z* ^−^)^~^; that is, *x*⊙*z* ≤ *y*⊙*z*. Similarly, we have that *x* ≤ *y* implies that *z*⊙*x* ≤ *z*⊙*y*.

(38) By (36), *x*⊙(*x*⇝*y*) ≤ *x*. By (35), *x*⊙(*x*⇝*y*) ≤ *y*. So, *x*⊙(*x*⇝*y*) ≤ *x*∧*y*. The second inequality follows similarly.

(39) Consider *x*⊙0 = (*x* → 0^−^)^~^ = (*x* → 1)^~^ = 1^~^ = 0,0⊙*x* = (0 → *x* ^−^)^~^ = 1^~^ = 0.

(40) For any *u*, *x*⊙(⋁_*i*∈*I*_ *x*_*i*_) ≤ *u*⇔⋁_*i*∈*I*_ *x*_*i*_ ≤ *x*⇝*u*⇔*x*_*i*_ ≤ *x*⇝*u* for each *i* ∈ *I*⇔*x*⊙*x*_*i*_ ≤ *u* for each *i* ∈ *I*⇔⋁_*i*∈*I*_(*x*⊙*x*_*i*_) ≤ *u* for each *i* ∈ *I*. The second equality follows similarly.

(41) Consider (*x*⊙*y*) → *z* = (*x* → *y* ^−^)^~^ → *z* = *z* ^−^⇝(*x* → *y* ^−^) = *x* → (*z* ^−^⇝*y* ^−^) = *x* → (*y* → *z*). The second equality follows similarly.

(42) For any *z*, *z* ≤ *y*⇝(⋀_*i*∈*I*_ *x*_*i*_)⇔*y*⊙*z* ≤ ⋀_*i*∈*I*_ *x*_*i*_⇔*y*⊙*z* ≤ *x*_*i*_ for each *i* ∈ *I*⇔*z* ≤ *y*⇝*x*_*i*_ for each *i* ∈ *I*⇔*z* ≤ ⋀_*i*∈*I*_(*y*⇝*x*_*i*_). The second equality follows similarly.

(43) Similarly.

(44) Consider *x*⊙*x* ^~^ = (*x* → *x* ^~−^)^~^ = 1^~^ = 0, *x* ^−^⊙*x* = (*x* ^−^ → *x* ^−^)^~^ = 0.

(45) By (41), *x*⊙*y*⇝*x* = *y*⇝(*x*⇝*x*) = 1 and *x*⊙*y* → *y* = *x* → (*y* → *y*) = 1. Hence, *x*⊙*y* ≤ *x*∧*y*.

(46) By (40), (*x*∨*y*)⊙(*x*∨*z*) = (*x*⊙*x*)∨(*x*⊙*z*)∨(*y*⊙*x*)∨(*y*⊙*z*) ≤ *x*∨*x*∨*x*∨(*y*⊙*z*) = *x*∨(*y*⊙*z*).

(47) By (13), (*x*⊙*z*)→(*y*⊙*z*) = (*x* → *z* ^−^)^~^ → (*y* → *z* ^−^)^~^ = (*y* → *z* ^−^)⇝(*x* → *z* ^−^) ≥ *x* → *y*. Similarly, we have the other one.

(48) By (35), *y*⊙(*y*⇝*z*) ≤ *z*. By (37), (*y*⊙(*y*⇝*z*))⊙*x* ≤ *z*⊙*x*; that is, *y*⊙((*y*⇝*z*)⊙*x*) ≤ *z*⊙*x*, and so (*y*⇝*z*)⊙*x* ≤ *y*⇝(*z*⊙*x*). The second inequality follows similarly.

**Proposition 13 .**

In a pseudo-*R*_0_ algebra, the following property holds:

(49)  (*x*⊙*y*)^−^∨((*x*∧*y*)→(*x*⊙*y*)) = (*y*⊙*x*)^~^∨((*x*∧*y*)⇝(*y*⊙*x*)) = 1.

**Proof**

By (pR1), (5), and (pR6),

(5) (x⊙y)−∨((x∧y)⟶(x⊙y)) =(x⟶y−)∨((x∧y)⟶(x⟶y−)~) =(x⟶y−)∨((x⟶y−)⇝(x∧y)−) =(x⟶y−)∨((x⟶y−)⇝(x−∨y−)) =1,(y⊙x)~∨((x∧y⇝(y⊙x)) =(x⇝y~)∨((x∧y)⇝(x⇝y~)−) =(x⇝y~)∨((x⇝y~)⟶(x∧y)~) =(x⇝y~)∨((x⇝y~)⟶(x~∨y~)) =1.

**Theorem 14 .**

Let A be a pseudo-*R*_0_ algebra. If A is a chain, then the operations → and ⇝ can be written by the following:

(6) $$\begin{array}{c} x⟶y=\{\begin{array}{cc} 1, & x\leq y,\\ x^{-}\vee y, & otherwise, \end{array}\\ x⇝y=\{\begin{array}{cc} 1, & x\leq y,\\ x^{\sim }\vee y, & otherwise. \end{array} \end{array}$$

**Proof**

If *x* ≤ *y*, by (4), *x* → *y* = *x*⇝*y* = 1. Now, we show that if *x*≰*y*, then *x* → *y* = *x* ^−^∨*y* and *x*⇝*y* = *x* ^~^∨*y*. By (12), we have *x* ^−^∨*y* ≤ *x* → *y* and *x* ^~^∨*y* ≤ *x*⇝*y*. On the other hand, by (pR6), we have

(7) (x⟶y)∨((x⟶y)⇝(x−∨y))  =(x⇝y)∨((x⇝y)⟶(x~∨y))=1.

Since *x*≰*y*, *x* → *y* ≠ 1 and *x*⇝*y* ≠ 1. By *A* being a chain, we have (*x* → *y*)⇝(*x* ^−^∨*y*) = 1 and (*x*⇝*y*)→(*x* ^~^∨*y*) = 1, and so *x* → *y* ≤ *x* ^−^∨*y* and *x*⇝*y* ≤ *x* ^~^∨*y*.

## 4. Categorical Equivalents to Pseudo-IMTL Algebras and Pseudo-NM Algebras

We show that pseudo-weak-*R*_0_ algebras coincide with pseudo-IMTL algebras and that pseudo-*R*_0_ algebras coincide with pseudo-NM algebras. Hence, as the corollaries of commutative cases, weak-*R*_0_ algebras coincide with IMTL-algebras and *R*_0_-algebras coincide with NM-algebras, which are obtained in [18].

**Theorem 15 .**

(i) Let *𝒜* = (*A*, ∨_*R*_, ∧_*R*_, →_*R*_, ⇝_*R*_,^−_*R*_^,^~_*R*_^, 0, 1) be a pseudo-weak-*R*_0_ algebra. Define Φ(*𝒜*) = (*A*, ∨_*I*_, ∧_*I*_, ⊙_*I*_, →_*I*_, ⇝_*I*_, 0,1) by

(8) $$\begin{array}{c} x\vee_{I}y=x\vee_{R}y,\;\;x\wedge_{I}y=x\wedge_{R}y,\\ x{⊙}_{I}y={\left(x{⟶}_{R}y^{{-}_{R}}\right)}^{\sim_{R}}={\left(y{⇝}_{R}x^{\sim_{R}}\right)}^{{-}_{R}},\\ x{⟶}_{I}y=x{⟶}_{R}y,\;\;x{⇝}_{I}y=x{⇝}_{R}y, \end{array}$$

where *x* ^−_*R*_^ = *x*→_*R*_0, *x* ^~_*R*_^ = *x*⇝_*R*_0. Then, Φ(*𝒜*) is a pseudo-IMTL algebra.

(ii) Conversely, let *𝒜* = (*A*, ∨_*I*_, ∧_*I*_, ⊙_*I*_, →_*I*_, ⇝_*I*_, 0,1) be a pseudo-IMTL algebra. Define Ψ(*𝒜*) = (*A*, ∨_*R*_, ∧_*R*_, →_*R*_, ⇝_*R*_,^−_*R*_^,^~_*R*_^, 0,1) by

(9) $$\begin{array}{c} x\vee_{R}y=x\vee_{I}y,\;\;x\wedge_{R}y=x\wedge_{I}y,\\ x^{{-}_{R}}=x{⟶}_{I}0,\;\;x^{\sim_{R}}=x{⇝}_{I}0,\\ x{⟶}_{R}y=x{⟶}_{I}y,\;\;x{⇝}_{R}y=x{⇝}_{I}y. \end{array}$$

Then, Ψ(*𝒜*) is a pseudo-weak-*R*_0_ algebra.

(iii) The above defined maps Φ and Ψ are mutually inverse.

**Proof**

(i) (pB1) is verified by (pL1).

(pB2) is verified by (32) and (33).

(pB3) is verified by (34).

(pB4) is verified by (18).

(pB5) is verified by (pL3).

Thus, Φ(*𝒜*) is a pseudo-IMTL algebra by Definition 3.

(ii) For convenience, in the following proof, we omit the indices “*R*” and “*I*” of the operations.

(pL1): By (pB1).

(pL2): If *x* ≤ *y*, by (pB3), *x* ≤ *y* ≤ *z* → (*y*⊙*z*) and *x* ≤ *y* ≤ *z*⇝(*z*⊙*y*), and so

$$\begin{array}{cc} \left(\text{a}\right) & x⊙z\leq y⊙z,\;\;z⊙x\leq z⊙y. \end{array}$$

If *x* ≤ *y*, by *(a)* and (pB3), (*y* → *z*)⊙*x* ≤ (*y* → *z*)⊙*y* and *x*⊙(*y*⇝*z*) ≤ *y*⊙(*y*⇝*z*) ≤ *z*, and so

$$\begin{array}{cc} \left(\text{b}\right) & y⟶z\leq x⟶z,\;\;y⇝z\leq x⇝z. \end{array}$$

Thus, if *x* ≤ *y*, by *(b)*, *y* → 0 ≤ *x* → 0 and *y*⇝0 ≤ *x*⇝0; that is, *y* ^−^ ≤ *x* ^−^ and *y* ^~^ ≤ *x* ^~^, which means that (pL2) holds.

(pL3): By (pB5).

(pR1): By (pB2) and (pB3), *x*⊙((*x*⇝*y*)⊙(*y*⇝*z*)) = (*x*⊙(*x*⇝*y*))⊙(*y*⇝*z*) ≤ *y*⊙(*y*⇝*z*) ≤ *z* and ((*y* → *z*)⊙(*x* → *y*))⊙*x* = (*y* → *z*)⊙((*x* → *y*)⊙*x*)≤(*y* → *z*)⊙*y* ≤ *z*, and so

(c)x⇝y≤(y⇝z)⟶(x⇝z),x⟶y≤(y⟶z)⇝(x⟶z).

Thus, by *(c)*, *x* → *y* ≤ (*y* → 0)⇝(*x* → 0) = *y* ^−^⇝*x* ^−^ ≤ *x* ^−~^ → *y* ^−~^ = *x* → *y* and *x*⇝*y* ≤ (*y*⇝0)→(*x*⇝0) = *y* ^~^ → *x* ^~^ ≤ *x* ^~−^⇝*y* ^~−^ = *x*⇝*y*, which means that *x* → *y* = *y* ^−^⇝*x* ^−^ and *x*⇝*y* = *y* ^~^ → *x* ^~^.

(pR2): By (pB2), *x*⊙1 = *x* ≤ *x* and 1⊙*x* = *x* ≤ *x*, and, by (pB3), *x* ≤ 1 → *x* and *x* ≤ 1⇝*x*. On the other hand, 1 → *x* = (1 → *x*)⊙1 ≤ *x* and 1⇝*x* = 1⊙(1⇝*x*) ≤ *x*. Hence, 1 → *x* = *x* and 1⇝*x* = *x*.

(pR3): By (pB2), (pB3), and *(a)*, (*z*⊙(*z*⇝*x*))⊙(*x*⇝*y*) ≤ *x*⊙(*x*⇝*y*) ≤ *y*; that is, *z*⊙((*z*⇝*x*)⊙(*x*⇝*y*)) ≤ *y*, so (*z*⇝*x*)⊙(*x*⇝*y*) ≤ *z*⇝*y*; hence, *x*⇝*y* ≤ (*z*⇝*x*)⇝(*z*⇝*y*). Similarly, (*x* → *y*)⊙((*z* → *x*)⊙*z*)≤(*x* → *y*)⊙*x* ≤ *y*; that is, ((*x* → *y*)⊙(*z* → *x*))⊙*z* ≤ *y*, so (*x* → *y*)⊙(*z* → *x*) ≤ *z* → *y*; hence, *x* → *y* ≤ (*z* → *x*)→(*z* → *y*).

(pR4): By (pB2), (pB3), and *(a)*, (*y*⊙(*x* → (*y*⇝*z*)))⊙*x* = *y*⊙((*x* → (*y*⇝*z*))⊙*x*) ≤ *y*⊙(*y*⇝*z*) ≤ *z*, and so *y*⊙(*x* → (*y*⇝*z*)) ≤ *x* → *z*; it follows that *x* → (*y*⇝*z*) ≤ *y*⇝(*x* → *z*). Similarly, we have *y*⇝(*x* → *z*) ≤ *x* → (*y*⇝*z*). Hence, *y*⇝(*x* → *z*) = *x* → (*y*⇝*z*).

(pR5): If *x* ≤ *y*, by (pB3), *z*⊙(*z*⇝*x*) ≤ *x* ≤ *y* and (*z* → *x*)⊙*z* ≤ *x* ≤ *y*, and so

$$\begin{array}{cc} \left(\text{d}\right) & z⇝x\leq z⇝y,\;\;z⟶x\leq z⟶y. \end{array}$$

Since *y* → *z* ≤ *y* → *z* and (*y* → *z*)⊙*z* ≤ *z*, by (pB3), *y* ≤ (*y* → *z*)⇝*z* and *z* ≤ (*y* → *z*)⇝*z*, and so *y*∨*z* ≤ (*y* → *z*)⇝*z*. Hence, by *(d)* and (pR4), *x* → (*y*∨*z*) ≤ *x* → ((*y* → *z*)⇝*z*) = (*y* → *z*)⇝(*x* → *z*), so (*y* → *z*)⊙(*x* → (*y*∨*z*)) ≤ *x* → *z*. Similarly, (*x*⇝(*y*∨*z*))⊙(*y*⇝*z*) ≤ *x*⇝*z*. Namely,

(e)(y⟶z)⊙(x⟶(y∨z))≤x⟶z,(x⇝(y∨z))⊙(y⇝z)≤x⇝z.

By (pB3) and the same proof of (40), we have

(f)x⊙(⋁i∈Ixi)  =⋁i∈I(x⊙xi),(⋁i∈Ixi)⊙x=⋁i∈I(xi⊙x),

whenever the arbitrary unions exist.

By (pB2), (pB4), *(f)*, and *(e)*, *x* → (*y*∨*z*) = 1⊙(*x* → (*y*∨*z*)) = ((*y* → *z*)∨(*z* → *y*))⊙(*x* → (*y*∨*z*)) = ((*y* → *z*)⊙(*x* → (*y*∨*z*))∨((*z* → *y*))⊙(*x* → (*y*∨*z*))≤(*x* → *z*)∨(*x* → *y*). On the other hand, by *(d)*, *x* → (*y*∨*z*) ≥ *x* → *y* and *x* → (*y*∨*z*) ≥ *x* → *z*, and so *x* → (*y*∨*z*)≥(*x* → *y*)∨(*x* → *z*). Hence, *x* → (*y*∨*z*) = (*x* → *y*)∨(*x* → *z*). Similarly, we have *x*⇝(*y*∨*z*) = (*x*⇝*y*)∨(*x*⇝*z*). Namely, (pR5) holds.

Thus, Ψ(*𝒜*) is a pseudo-weak-*R*_0_ algebra by Definition 8.

(iii) We put the index “c” to the operations of the structure obtained by composition of Φ and Ψ.

Let *𝒜* = (*A*, ∨_*R*_, ∧_*R*_, →_*R*_, ⇝_*R*_, 0,1) be a pseudo-weak-*R*_0_ algebra. We prove that Ψ(Φ(*𝒜*)) = *𝒜*. Indeed,*x*∨_*c*_ *y* = *x*∨_*I*_ *y* = *x*∨_*R*_ *y*, *x*∧_*c*_ *y* = *x*∧_*I*_ *y* = *x*∧_*R*_ *y*, *x* ^−_*c*_^ = *x*→_*I*_0 = *x*→_*R*_0, *x* ^~_*c*_^ = *x*⇝_*I*_0 = *x*⇝_*R*_0, *x*→_*c*_ *y* = *x*→_*I*_ *y* = *x*→_*R*_ *y*, and *x*⇝_*c*_ *y* = *x*⇝_*I*_ *y* = *x*⇝_*R*_ *y*.

Let *𝒜* = (*A*, ∨_*I*_, ∧_*I*_, ⊙_*I*_, →_*I*_, ⇝_*I*_, 0,1) be a pseudo-IMTL algebra. We prove that Φ(Ψ(*𝒜*)) = *𝒜*. Indeed, *x*∨_*c*_ *y* = *x*∨_*R*_ *y* = *x*∨_*I*_ *y*, *x*∧_*c*_ *y* = *x*∧_*R*_ *y* = *x*∧_*I*_ *y*, *x*⊙_*c*_ *y* = (*x*→_*R*_ *y* ^−_*R*_^)^~_*R*_^ = (*x*→_*I*_ *y* ^−_*I*_^)^~_*I*_^ = *x*⊙_*I*_ *y*, *x*→_*c*_ *y* = *x*→_*R*_ *y* = *x*→_*I*_ *y*, and*x*⇝_*c*_ *y* = *x*⇝_*R*_ *y* = *x*⇝_*I*_ *y*.

**Theorem 16 .**

(i) Let *𝒜* = (*A*, ∨_*R*_, ∧_*R*_, →_*R*_, ⇝_*R*_, ^−_*R*_^, ^~_*R*_^, 0,1) be a pseudo-*R*_0_ algebra. Define Φ(*𝒜*) = (*A*, ∨_*I*_, ∧_*I*_, ⊙_*I*_, →_*I*_, ⇝_*I*_, 0,1) by

(10) $$\begin{array}{c} x\vee_{I}y=x\vee_{R}y,\;\;x\wedge_{I}y=x\wedge_{R}y,\\ x{⊙}_{I}y={\left(x{⟶}_{R}y^{{-}_{R}}\right)}^{\sim_{R}}={\left(y{⇝}_{R}x^{\sim_{R}}\right)}^{{-}_{R}},\\ x{⟶}_{I}y=x{⟶}_{R}y,\;\;x{⇝}_{I}y=x{⇝}_{R}y, \end{array}$$

where *x* ^−_*R*_^ = *x*→_*R*_0, *x* ^~_*R*_^ = *x*⇝_*R*_0. Then, Φ(*𝒜*) is a pseudo-NM algebra.

(ii) Conversely, let *𝒜* = (*A*, ∨_*I*_, ∧_*I*_, ⊙_*I*_, →_*I*_, ⇝_*I*_, 0,1) be a pseudo-NM algebra. Define Ψ(*𝒜*) = (*A*, ∨_*R*_, ∧_*R*_, →_*R*_, ⇝_*R*_,^−_*R*_^,^~_*R*_^, 0,1) by

(11) $$\begin{array}{c} x\vee_{R}y=x\vee_{I}y,\;\;x\wedge_{R}y=x\wedge_{I}y,\\ x^{{-}_{R}}=x{⟶}_{I}0,\;\;x^{\sim_{R}}=x{⇝}_{I}0,\\ x{⟶}_{R}y=x{⟶}_{I}y,\;\;x{⇝}_{R}y=x{⇝}_{I}y. \end{array}$$

Then, Ψ(*𝒜*) is a pseudo-*R*_0_ algebra.

(iii) The above defined maps Φ and Ψ are mutually inverse.

**Proof**

(i) By Theorem 15 (i), (pB1)–(pB5) hold in Φ(*𝒜*). (pB6) is verified by (49).

(ii) By Theorem 15 (ii), (pL1)–(pL3) and (pR1)–(pR5) hold in Ψ(*𝒜*). Now, we prove that (pR6) also holds in Ψ(*𝒜*). For convenience, in the following proof, we omit the indices “*R*" and “*I*” of the operations.

By (pB2) and (pB3), for any *u* ∈ *A*, *u* ≤ *x* → (*y* → *z*)⇔*u*⊙*x* ≤ *y* → *z*⇔*u*⊙(*x*⊙*y*) = (*u*⊙*x*)⊙*y* ≤ *z*⇔*u* ≤ (*x*⊙*y*) → *z* and *u* ≤ *x*⇝(*y*⇝*z*)⇔*x*⊙*u* ≤ *y*⇝*z*⇔(*y*⊙*x*)⊙*u* = *y*⊙(*x*⊙*u*) ≤ *z*⇔*u* ≤ (*y*⊙*x*)⇝*z*. Hence,

(g)(x⊙y)⟶z=x⟶(y⟶z),(y⊙x)⇝z=x⇝(y⇝z).

By *(g)* and (pR4), (*x*⊙*y*)^~^ = (*x*⊙*y*)⇝0 = *y*⇝(*x*⇝0) = *y*⇝(1 → *x* ^~^) = *y*⇝*x* ^~^. Similarly, (*x*⊙*y*)^−^ = (*x*⊙*y*) → 0 = *x* → (*y* → 0) = *x* → (1⇝*y* ^−^) = *x* → *y* ^−^. Hence,

$$\begin{array}{cc} \left(\text{h}\right) & x⊙y={\left(y⇝x^{\sim }\right)}^{-}={\left(x⟶y^{-}\right)}^{\sim }. \end{array}$$

By (pL2), (pL3), and the same proof of (5) and (6), we have

(i)(x∧y)~=x~∨y~,    (x∧y)−=x−∨y−,(x∨y)~=x~∧y~,    (x∨y)−=x−∧y−.

Thus, by *(h)*, *(i)*, and (pB6), we have

(12) (x⟶y)∨((x⟶y)⇝(x−∨y))  =(x⟶y)∨((x−∨y)~⟶(x⟶y)~)  =(x⟶y~−)~−∨((x−∨y)~⟶(x⟶y~−)~)  =(x⊙y~)−∨((x∧y~)⟶(x⊙y~))  =1,(x⇝y)∨((x⇝y)⟶(x~∨y))  =(x⇝y)∨((x~∨y)−⇝(x⇝y)−)  =(x⇝y−~)−~∨((x∧y−)⇝(x⇝y−~)−)  =(y−⊙x)~∨((x∧y−)⇝(y−⊙x))  =1.

(iii) By Theorem 15 (iii).

In Theorems 15 and 16, if →_*R*_ = ⇝_*R*_, ^−_*R*_^=^~_*R*_^, and →_*I*_ = ⇝_*I*_, then we have the main results in [18].

**Corollary 17 (see [18, Theorem 6]).**

Weak-*R*_0_ algebras coincide with IMTL-algebras.

**Corollary 18 (see [18, Theorem 5]).**

*R*_0_-algebras coincide with NM-algebras.

## 5. Conclusions

We gave a positive answer to Iorgulescu's open problem. We extended weak-*R*_0_ algebras and *R*_0_-algebras to the noncommutative forms, called pseudo-weak-*R*_0_ algebras and pseudo-*R*_0_ algebras. The properties of pseudo-weak-*R*_0_ algebras and pseudo-*R*_0_ algebras were investigated, and the simplified axiom systems of pseudo-weak-*R*_0_ algebras and pseudo-*R*_0_ algebras were discussed. Finally, we showed that pseudo-weak-*R*_0_ algebras are categorically isomorphic to pseudo-IMTL algebras and that pseudo-*R*_0_ algebras are categorically isomorphic to pseudo-NM algebras. Based on these results, we will study filter theory of pseudo-weak-*R*_0_ algebras and pseudo-*R*_0_ algebras and investigate relations between various kinds of filters of pseudo-logic algebras. We may also study fuzzy type of filters of pseudo-weak-*R*_0_ algebras and pseudo-*R*_0_ algebras.

## References

[1] Imai Y, Iseki K (1966). On axiom system of propositional calculus. *Proceedings of the Japan Academy*.

[2] Georgescu G, Iorgulescu A Pseudo-BCK algebras: an extension of BCK algebras.

[3] Liu YL, Liu SY, Xu Y (2007). Pseudo-BCK algebras and PD-posets. *Soft Computing*.

[4] Chang CC (1958). Algebraic analysis of many valued logics. *Transactions of the American Mathematical Society*.

[5] Georgescu G, Iorgulescu A (2001). Pseudo MV algebras. *Multiple Valued Logic*.

[6] Hajek P (1998). *Metamathematics of Fuzzy Logic*.

[7] Georgescu G, Iorgulescu A Pseudo-BL algebras: a noncommutative extension of BL algebras.

[8] Di Nola A, Georgescu G, Iorgulescu A (2002). Pseudo-BL algebras: part I. *Multiple Valued Logic*.

[9] Di Nola A, Georgescu G, Iorgulescu A (2002). Pseudo-BL algebras: part II. *Multiple Valued Logic*.

[10] Zhang X-H, Fan X-S (2008). Pseudo-BL algebras and pseudo-effect algebras. *Fuzzy Sets and Systems*.

[11] Zhan JM, Jun YB, Kim HS (2014). Some types of falling fuzzy filters of BL-algebras and its applications. *Journal of Intelligent and Fuzzy Systems*.

[12] Esteva F, Godo L (2001). Monoidal t-norm based logic: towards a logic for left-continuous t-norms. *Fuzzy Sets and Systems*.

[13] Flondor P, Georgescu G, Iorgulescu A (2001). Pseudo-t-norms and pseudo-BL algebras. *Soft Computing*.

[14] Wang G (1997). A formal deductive system for fuzzy propositional calculus. *Chinese Science Bulletin*.

[15] Zhan JM, Pei DW, Jun YB (2013). Falling fuzzy (implicative) filters of *R*
_0_-algebras and its applications. *Journal of Intelligent and Fuzzy Systems*.

[16] Iorgulescu A (2006). Classes of pseudo-BCK algebras—part I. *Journal of Multiple-Valued Logic and Soft Computing*.

[17] Liu YL, Zhang XH (2006). Pseudo NM-algebras and their properties. *Chinese Journal of Engineering Mathematics*.

[18] Pei D (2003). On equivalent forms of fuzzy logic systems NM and IMTL. *Fuzzy Sets and Systems*.

